# Improving the Quality of Postpartum Care in Ghana: Protocol for a Parallel Randomized Controlled Trial

**DOI:** 10.2196/47519

**Published:** 2023-08-22

**Authors:** Yenupini Joyce Adams, John Stephen Agbenyo

**Affiliations:** 1 Eck Institute for Global Health University of Notre Dame Notre Dame, IN United States; 2 Savana Signatures Tamale Ghana

**Keywords:** postpartum care, postnatal care, maternal mortality, Ghana, randomized controlled trial, protocol

## Abstract

**Background:**

Although the postpartum period poses substantial risks and can result in significant maternal morbidity and mortality, postpartum care of the mother receives much less attention in transitional countries.

**Objective:**

We describe the protocol for a randomized controlled trial to implement and evaluate a postpartum care delivery model titled Focused-PPC (Focused Postpartum Care).

**Methods:**

Focused-PPC is an integrated group postpartum care model that meets the clinical care, education, and support needs of mothers up to 1 year after birth. The Focused-PPC intervention is a parallel randomized controlled trial with a total of 192 postpartum women at 4 health centers in Tamale, Ghana. Participants will be randomized into 1 of 2 trial arms at a 1:1 allocation ratio: (1) the control arm, which receives the standard postnatal care currently delivered in health facilities, or (2) the intervention arm, which receives the Focused-PPC model of care. Women enrolled in the intervention arm will receive postpartum clinical assessments and education for the first 6 weeks and will continue to receive education, measures of vital signs, and peer support for 12 months post partum during child welfare visits. Led by trained midwives, each postpartum group in the intervention arm will meet at 1-2 weeks, 6 weeks, and monthly thereafter for up to 1 year post partum, following the Ghana Health Service postnatal care schedule.

**Results:**

The Focused-PPC guide, data collection tools, and audiovisual education materials were successfully developed and translated into the local language. We have enrolled and conducted baseline surveys for 192 women (sample size met) in the Focused-PPC trial who have been randomized into intervention and control arms. We have established a total of 12 Focused-PPC groups in the intervention arm, 3 groups from each site, all of which have sessions underway.

**Conclusions:**

Focused-PPC has the potential to change the postpartum care delivery model in Ghana and other countries in sub-Saharan Africa and beyond.

**Trial Registration:**

ClinicalTrials.gov NCT05280951; https://clinicaltrials.gov/study/NCT05280951

**International Registered Report Identifier (IRRID):**

DERR1-10.2196/47519

## Introduction

Maternal mortality is a major global health challenge, defined as the death of a woman while, or up to 42 days after, termination of pregnancy, related to the pregnancy itself and not by accidental causes [1]. Around the world, about 810 women die every day from preventable causes related to pregnancy and childbirth [1]. Most maternal deaths are preventable, yet sub-Saharan Africa continues to be impacted disproportionately compared to the rest of the globe, accounting for two-thirds (66%) of all maternal mortality worldwide [1]. Ghana has a particularly high maternal mortality ratio of 308 deaths per 100,000 live births, compared to a maternal mortality ratio of 211 globally and only 11 in high-income countries [1]. Most of these deaths occur during the postpartum period, the period following birth, as compared to during pregnancy or labor and delivery [2].

In terms of health care provision, the postpartum period receives much less attention than pregnancy or childbirth. However, the period after birth still poses substantial risk to mothers and can result in significant maternal morbidity and mortality [1]. The median national coverage of routine postnatal visits for the mother stands at 71%, which is still significantly behind global targets for 2025, with some nations demonstrating coverage as low as 44% [3]. The World Health Organization (WHO) recommendations for postpartum care include continuous care in a health care facility for up to 24 hours and further postpartum clinical examinations at 48-72 hours, 7-14 days, and 6 weeks after birth [4]. These recommendations also state that postpartum clinical examinations should include assessments of vital signs, vaginal bleeding or discharge, uterine contraction, fundal height, urine void, and breast tenderness [4]. However, within developing countries, recommended postpartum clinical assessments are not consistently provided to women at recommended time points [5,6]. For instance, among women delivering in health facilities in Malawi, over half reported a lack of postpartum clinical assessments, including blood pressure checks, temperature checks, abdominal examinations, vaginal examinations, and breast examinations [5].

Mortality is worsened by a lack of access to appropriate postpartum care. Postpartum complications are frequently unforeseen and necessitate an immediate reaction, making quality care essential for the health and survival of the mother after birth. For instance, postpartum hemorrhage can be fatal within a matter of hours without prompt treatment [7]. Sufficient postpartum care enables medical professionals to quickly recognize and address complications as they arise [8]. Postpartum care also provides the opportunity for health care providers to meet the social and health needs of women and to encourage women to adopt evidence-based postpartum practices at home. As maternal self-care typically occurs at home after birth, it is essential that mothers are empowered with proper knowledge to mitigate their health risks after birth [4].

Within the postpartum period, obstetric complications are the primary cause of direct maternal deaths worldwide [2]. Complications such as hemorrhage, hypertension, and sepsis account for 62.4% of maternal deaths in sub-Saharan Africa [9]. Each of these complications has identifiable warning signs that, if noticed early, are likely to result in improved maternal outcomes. Therefore, ensuring knowledge of warning signs of complications is a key strategy in improving women’s complication readiness and reducing deaths resulting from delays in seeking care [10]. If a mother has adequate knowledge of warning signs, she is far more likely to promptly recognize symptoms and seek timely postpartum care. However, several studies have reported that postpartum women in transitional countries experience inadequate knowledge of postpartum warning signs of complications [11-14]. Furthermore, midwives working in Tamale, Ghana, have self-reported inadequate knowledge and skill sets to adequately educate patients about all detrimental postpartum complications and how to manage those complications [15,16].

Comprehensive patient education is a widely established, low-cost intervention effective in improving health outcomes [17-19]. Patient education addressing postpartum recovery and potential complications that may happen after birth has significant potential to improve postpartum health outcomes among women in Ghana. Women are expected to manage their postpartum recovery following discharge from the hospital, meaning that all women are expected to recognize potential complications and seek timely emergency care accordingly from 48 hours after birth and beyond [20]. Therefore, it is crucial to ensure that all women, regardless of their risk factors, are educated regarding the early warning signs of potential complications and when symptoms necessitate care-seeking. Maternal morbidity and mortality can be reduced through the provision of quality education administered by health care providers [21].

From our experience working in the setting, there is a lack of integrated postpartum care that incorporates recommended clinical assessments, education, and support for women after birth. Our intervention, entitled Focused Postpartum Care (Focused-PPC), fills this gap through the provision of comprehensive postpartum clinical care, targeted education, and peer support for women in Ghana. A shift toward standardized care simultaneously addressing the needs of both the mother and the baby has significant potential to reduce maternal morbidity and mortality in this setting. In this paper, we describe the protocol for a randomized controlled trial in Tamale, Ghana. The aim of our study is to implement and evaluate Focused-PPC in a parallel randomized controlled trial with 192 postpartum women at 4 health centers in Tamale, Ghana.

## Methods

### Study Design

This is a parallel randomized controlled trial with balanced randomization (1:1) conducted in 4 health centers in Sagnarigu municipality, Tamale, Ghana. An overview of the study design can be found in Textbox 1*.*

**Study design.**

- Recruitment of potential participants
- Enrollment of eligible participants
- Informed consent and baseline survey
- Random assignment into intervention and control groups:
  Kanvilli: 48 participants (intervention: n=24 [3 groups of 8]; control: n=24)
  Choggu: 48 participants (intervention: n=24 [3 groups of 8]; control: n=24)
  Kalpohin: 48 participants (intervention: n=24 [3 groups of 8]; control: n=24)
  Bagabaga: 48 participants (intervention: n=24 [3 groups of 8]; control: n=24)
- Focused Postpartum Care (Focused-PPC; intervention arm)
  Focused-PPC care
  Focus group discussions at end
- Standard postnatal care (control arm)
  Usual care
  Not applicable
- Interviewer administered surveys at certain time points (see Table 1) for both arms

**Table 1:** Outcomes and evaluation.

Variables	Measurement	Baseline	1-2 weeks	6 weeks	3 months	6 months	12 months
Independent variables and potential covariates	Demographic questions, Obstetric history questions, Readiness for Hospital Discharge Scale – New Mother Form	✓					
Knowledge of postbirth warning signs	Investigator developed questions based on common postbirth complications	✓	✓		✓	✓	
Postpartum health behaviors	Specific questions assessing health behaviors will be tailored to the time frame post partum. Will include attendance to visits		✓	✓	✓	✓	✓
Postpartum health status	Edinburgh Postnatal Depression Scale, Perceived Stress Scale, Questions on complications experienced and other questions on health status as appropriate to the time frame after delivery, Measures of blood pressure, temperature, pulse, and weight	✓	✓	✓	✓	✓	✓
Feasibility, acceptability, and satisfaction	Focus groups with midwives and each group of participants in Focused-PPC^a^ intervention arm using focus group discussion guide						At exit

^a^Focused-PPC: Focused Postpartum Care.

### Setting

The study will be conducted in Sagnarigu municipality, Tamale, which is located in the Northern region of Ghana. The public health sector in this area is run by the Ghana Health Service (GHS) and constitutes the most commonly accessed health facilities. The study will be conducted at 4 GHS health centers. The health centers are: Choggu Health Center, Kanvilli Health Center, Bagabaga Health Center, and Kalpohin Health Center.

### Participants and Recruitment

Participants who are 18 years of age or older will be recruited in their third trimester while attending antenatal care visits in the health centers. Project assistants employed in each health center will conduct face-to-face recruitment using a script. The script describes the study, its eligibility criteria, and its voluntary nature. Typically, women arrive at the antenatal care visit and wait until it is their turn to enter the consulting room. Recruitment will occur during this wait time. When eligible patients agree to participate, they will provide their name and contact information for follow-up. Eligible and interested participants will also be provided with the project contact information and project assistant number to call or text at delivery, and project assistants will also follow up with women. Participants will be enrolled in the research study after delivery. Eligibility criteria at enrollment include being currently admitted to the postnatal ward, having had a live birth, having a newborn not in the neonatal intensive care unit, being able to speak and understand English or Dagbani, and having the capacity to provide informed consent. Since the intervention arm requires participants to have given birth within the same week or 2, randomization into the intervention arm (Focused-PPC) and control arm (usual care) will occur after each woman has given birth.

### Sample Size

To establish an appropriate sample size, a power analysis was conducted using G*Power (Erdfelder, Faul, and Buchner) [22,23]. The experimental design has 4 total health centers. Individuals at every health center will be randomly assigned to an intervention arm, keeping in mind that the intervention arm needs to be in multiples of 8 at a time and that intervention groups are equal in size. Baseline characteristics are not expected to affect the outcome. In G*Power, the power analysis was run with 2 groups (the control arm and the intervention arm), which were then split up into 8-equal sized subgroups (health center A intervention, health center A control, ...., health center D intervention, health center D control). The model included 3 time points for measurements. The correlation among repeated measures that varied for a range of values from 0.05 (representing nearly uncorrelated measurements) to 0.3 (representing moderately correlated measurements) was considered. The desired power was always set to 0.8, and the nominal type I error was set to .05. A range of Cohen (1988) effect sizes was evaluated, from 0.10 (very small) up to 0.25 (a medium effect size). The outcome, the mean knowledge of postbirth warning signs, is a continuous variable. This was accomplished by varying the intervention arm’s mean knowledge level. Based on previous study data, 1.9 was used as the control sample’s mean knowledge score, and 1.4 was used as the SD within groups. For the intervention sample, a range of values for the mean knowledge score was tested, from 2.2 (which equates to a 0.1 effect size) to 2.65 (equating to a 0.25 effect size). The control arm and the intervention arm were assumed to have the same SD.

Under optimal conditions, where the correlation among repeated measures is minimal at 0.05 with a medium effect size of 0.25, a total sample size of 64 (16 individuals split into 1 intervention and 1 control group per clinic) is required [24]. Whereas under the worst assumed conditions (a small effect size of 0.10 and any correlation among repeated measures), a total sample size of 512 (128 individuals per clinic split into 16 intervention and 16 control groups) would be necessary. Assuming we want to detect a Cohen f of 0.15 with any size correlation up to 0.3 among repeated measures and no attrition, the recommended sample size is 192 (with 48 individuals split up into 3 intervention and 3 control groups per clinic).

### Randomization

Project assistants will meet with eligible participants after birth, at discharge, in a designated private spot at the health facility, where they will obtain consent and complete the baseline survey. After the baseline survey, participant allocation will be 1:1 for equal intervention and control sizes. Allocation will be completed according to a computer-generated list of random numbers, allocating group sizes of 8. The allocation will be generated by a data scientist on the research team. The data scientist will provide participant allocations (intervention arm or control arm) to the project team. Participants in the intervention arm will also receive a group allocation (allocated in groups of 8). Intervention arm group participants will then be contacted to begin their group postpartum care. There will be no blinding, as the study team will know which women are in the intervention arm (receiving Focused-PPC) and the control arm (receiving usual care).

### Group Conditions

#### Control Arm

Women in the control arm will receive standard postnatal care currently practiced in their respective health centers. This involves attending visits at the health center within 14 days after birth, at 6 weeks, and monthly thereafter up to 1 year. Currently, the GHS recommends beginning postnatal care immediately after birth, with the mother and baby receiving care within 14 days and at 6 weeks. After 6 weeks, the mother attends monthly child welfare clinic visits with the baby for up to 12 months.

#### Intervention Arm

##### Overview

Each Focused-PPC group in the intervention arm will consist of 8 postpartum women who will meet at 1-2 weeks, 6 weeks, and monthly thereafter for up to 1 year post partum, following the GHS postnatal care schedule. All Focused-PPC group sessions follow a guide developed by the research team with content derived from evidence-based guidelines, reviewed by experts, and approved by the GHS. These group sessions consist of two components: (1) 15-minute individual clinical assessments and counseling for each mother performed by midwives and (2) a 1-hour-long group education session and peer support led by midwives. Each Focused-PPC session will be led by 2 trained and registered midwives from each health center, who will provide both clinical care and education and support as needed. In addition, each group session will be supported by a project assistant, who will be responsible for collecting research data. Focused-PPC will be implemented in addition to newborn care.

##### Postpartum Clinical Assessments

The clinical care component of the intervention follows the recommended clinical assessments by the GHS and the WHO and includes assessment of vital signs, fundal assessment, vaginal bleeding or discharge assessment, incision assessment, and a breast examination. After the 6-week postpartum time point, only vital signs are measured at each session. In sub-Saharan Africa, over 60% of maternal deaths occur within the vulnerable first year after birth [2]. Since maternal deaths extend far beyond the traditional 6-week time frame, it is important that quality clinical care extend to 1 after birth. Focused-PPC provides regular contact with and monitoring by a midwife for up to a year following birth.

##### Postpartum Education

The content of Focused-PPC education will focus on the unique needs of postpartum women based on the time frame after delivery. Educational resources will be developed by the research team and an expert panel to apply to different time points throughout the postpartum period and to address the complex needs of postpartum mothers while conforming to the regulations and practices of the GHS. Educational content will then be converted into audiovisual resources, which will be used by midwives to present information during Focused-PPC sessions. Education content will cover the following topics: what to expect for normal postdelivery recovery, self-care, potential postpartum complications and danger signs, risk factors, food and nutrition, breastfeeding, mental health, social support, family planning, menstrual cycles, and interconception care.

##### Postpartum Peer Support

Women in Focused-PPC groups will engage in peer support by discussing issues relevant to them. Group prenatal care models have been shown to improve both pregnancy outcomes and maternal satisfaction with care [25-27]. In the postpartum period, group treatment and peer support programs have been shown to improve postpartum depression and prevent postpartum depression [28-31].

#### Intervention Fidelity

The Focused-PPC intervention will be maintained and monitored through fidelity checklists that are specific to each time point at which a session is held. These checklists will be completed by project assistants and midwives at every session. Additionally, video recordings will be recorded during Focused-PPC sessions at the initial sessions, midpoint sessions, and final sessions. Project assistants will also file session reports that address participant actions and discussions and other session details.

### Data Collection or Measures

In addition to the baseline survey, all participants will be given surveys at 1-2 weeks, 6 weeks, 3 months, 6 months, and 12 months from baseline. All surveys will be interviewer-administered by the project assistants and completed electronically via tablets using a secure database system. Strict procedures will be followed to protect participants’ confidentiality. We also plan to evaluate the feasibility, acceptability, and satisfaction of Focused-PPC. Focus Group Discussions will be conducted with the midwives who implemented Focused-PPC and with each group of participants enrolled in the Focused-PPC intervention arm after their 12-month sessions.

Independent variables and potential covariates will be measured through demographic and obstetric history questions and the Dagbani Readiness for Hospital Discharge Scale-New Mother Form (RHDS-NM). The RHDS-NM is a 22-item instrument that includes a self-reported rating scale with item scores ranging from 0 to 10 and possible scale scores ranging from 0 to 220 [32]. Higher scores indicate greater readiness for hospital discharge. The RHDS-NM, designed for use within English-speaking populations, was translated into Dagbani and adapted for use in Tamale. The Dagbani version of the RHDS-NM has demonstrated excellent reliability (Cronbach ɑ=.94) and measures postpartum mothers’ perceptions of readiness for discharge through examination of their personal status, knowledge, coping ability, and expected support [33].

The primary outcome is knowledge of postbirth warning signs, measured through questions developed based on an evidence-based assessment of the most common postbirth complications. The secondary outcome is postpartum health behaviors, including specific questions assessing health behaviors tailored to the specific time frame postpartum. Other descriptive outcomes include postpartum health status, which will include measures of postpartum depression, stress, complications experienced, vital signs, and other questions on health status as appropriate to the time frame after delivery. Postpartum depression is measured through the Edinburgh Postnatal Depression Scale (EPDS), a self-report screening tool commonly used in perinatal settings [34,35]. The EPDS consists of 10 items assessing women’s depressive symptoms over the past week. The EPDS has a maximum score of 30, with higher scores indicating higher frequency and increased severity of depressive symptoms. Stress is measured through the Perceived Stress Scale (PSS), a 10-item scale assessing women’s stress levels over the last month [36]. The PSS evaluates a woman’s perceptions of unpredictability, lack of control, and overloading in her life, with a higher score indicating higher levels of perceived stress. The feasibility and acceptability of the intervention will be measured using end-project focus group discussion data obtained from midwives and participants in the intervention arm. Outcomes and time points of data collection can be found in Table 1.

### Analysis Plan

Differences in outcomes between the intervention and control arms will be assessed. We expect that the intervention arm will have higher knowledge of postbirth warning signs than the control arm at every time point measured and that the intervention arm will have a larger increase in knowledge of postbirth warning signs between time points. We expect postpartum health behaviors to be different between the intervention and control arms.

All data will be reviewed for completeness and transferred to STATA (StataCorp) statistical software for analyses [37]. The primary variable of interest is knowledge of postbirth warning signs, measured on 4 occasions. The baseline measurement of knowledge of postbirth warning signs will be used in an initial *t* test to confirm that there is no difference in knowledge between the intervention and control arms before the study to validate the randomization procedure. We will model and test the difference between intervention and control arm knowledge of postbirth warning signs at each time point, and knowledge between time points will be assessed using a repeated measures multivariate ANOVA (MANOVA) with a time and treatment interaction as the primary analysis controlling for baseline demographic variables. For the intervention arm, follow-up secondary analyses will measure the change in postpartum health status, postpartum health behaviors, and clinical assessment data over time using repeated measures MANOVA. Ancillary analyses to be run between the intervention and control arms include a proportion test for the patient’s readiness for hospital discharge. Additionally, an evaluation of feasibility, acceptability, and satisfaction will be summarized for inclusion when discussing the cost or benefit of the intervention.

Using intent-to-treat, participants providing data at baseline and first check-in will be included. Missing data (eg, due to attrition) will be accounted for using multiple imputations via the mice package in R (R Core Team) [38]. Sensitivity to missing data will be assessed by comparing multiple imputations to complete case analyses.

### Ethics Approval

The Informed Consent process and all other study procedures were reviewed and approved by the University of Notre Dame institutional review board (protocol 21-06-6662). Additionally, in-country ethical approval was obtained from the Navrongo Health Research Center institutional review board (App/Focused-PPC/7/2021). Approval to conduct the study has also been obtained from both the regional and district levels of the GHS. All project team members have current human subject training. Informed consent will be obtained from all participants.

## Results

In the first 6 months of the project, we will complete start-up activities inclusive of inception meetings with GHS representatives at the district and regional levels, developing training guides and Focused-PPC education content, training project staff, and developing data collection tools. In the next 6 months of the project, we will recruit and enroll participants, begin Focused-PPC intervention sessions, and complete data collection. Focused-PPC intervention sessions and data collection will continue in year 2 of the project. End-line focus group discussions will be conducted with project participants and midwives when groups have completed their final intervention sessions at 12 weeks.

Ethical approval was successfully obtained from the Navrongo Health Research Center, and the appropriate approvals were obtained from the GHS. The Focused-PPC Guide (education and session guide) was successfully developed with input from GHS and evidence-based resources and underwent multiple levels of expert review to produce the final guide. All team members, midwives, and project assistants have been trained in the study protocol. Measures for data collection were successfully translated into Dagbani (the local language), and audiovisual resources for the sessions were developed and translated into Dagbani. Clinical trial registration is completed at ClinicalTrials.gov (NCT05280951).

We have enrolled and conducted baseline surveys for 192 women (sample size met) in the Focused-PPC trial who have been randomized into intervention and control arms. We have established a total of 12 Focused-PPC groups in the intervention arm, 3 groups from each site, all of which have sessions underway. Midwives are successfully conducting Focused-PPC sessions using the study’s established guide and protocol. The Focused-PPC intervention is maintained and monitored through fidelity checklists, which are specific to each time point and completed by project assistants and midwives at every session. Project assistants are also documenting sessions through photos, videos, and session reports, addressing participant actions, discussions, and collaboration.

## Discussion

### Principal Findings

The purpose of this study is to implement and evaluate a postpartum care model entitled Focused-PPC in a parallel randomized controlled trial with 192 postpartum women at 4 health centers in Tamale, Ghana. Focused-PPC sessions are underway in all participating health facilities. The primary outcome is knowledge of post-birth warning signs. We expect that the Focused-PPC group will have higher knowledge of post-birth warning signs than the control group at every time point measured and that the Focused-PPC group will have a larger increase in knowledge of postbirth warning signs between time points. We are also measuring postpartum health behaviors such as eating a healthy diet, uptake of family planning, breastfeeding, and so on. We expect that more participants in the Focused-PPC groups will eat a balanced diet most days of the week and uptake family planning compared to participants in the control groups. Additionally, we also expect mental health outcomes such as positive depression screens to be significantly lower over time in the Focused-PPC group compared to the control group. Other descriptive outcomes include postpartum health status, and we expect to capture and treat blood pressure cases during the course of the study.

### Challenges Encountered

A major hurdle the team faced early in the intervention, which impacted the scheduled engagements with the leadership of GHS, was the application and subsequent acquisition of institutional review board clearance from the Navrongo Health Research Center. The clearance was obtained after about 3 months of waiting time, and implementation could not commence unless proof of clearance was presented to the leadership of GHS in the Northern Region for permission to be given for the team to commence implementation. Delays in reviewing the Focused-PPC guide also contributed to a later than expected start of recruitment. The guide went through multiple levels of review, and reviews took longer at each stage than had been planned. In addition, the project team faced some difficulties finding context-specific audiovisual content to be used as supporting aids during the FPPC sessions. Finding and creating context-specific teaching aids took longer than expected. There have been no protocol modifications to the study so far.

We are consistently monitoring and improving upon data collection as necessary. For instance, following issues with inconsistent participant tracking numbers, our data scientist was able to install a mechanism into our survey software that double-checks each tracking number and will not allow remaining data to be documented until this has been addressed. We have also implemented solutions to address accidental missed data by alerting project assistants in real time if important information is missing, allowing them to review entered surveys before submission, and getting an email confirmation immediately after submitting a survey. Our monitoring efforts on this front have led to more accurate and comprehensive data so far and also improved the process of recording data for our project assistants. Furthermore, we have held monthly meetings with the project team, including all members from both the United States and Ghana. These meetings have helped substantially in prioritizing the goals of the trial and have allowed us to properly deal with obstacles as they arise.

### Anticipated Limitations

Due to the nature of the study described in this protocol, the intervention is not blinded. There may be a potential for contamination since the midwives implementing the study are used in the health centers. However, since the study is not blinded, different midwives provide care and education to women during postpartum visits if they are in the control group to minimize contamination. In addition, the study is being conducted in one municipality in Tamale, so generalizability will be limited. We hope to conduct a larger study in the future. Given that the team in Ghana has worked in the setting since 2009 and has an established relationship with the health centers and women in the communities, we do not anticipate major recruitment difficulties. However, retention of participants and some participants missing scheduled sessions may be a challenge. We will engage participants and call them individually to remind them of upcoming sessions. We will ensure that midwives recap sessions are comprehensive enough to ensure knowledge transfer to those who missed previous sessions.

### Dissemination Plan

To ensure that the results of this research impact practice and maximize the benefit to women, we will target multiple audiences with our research findings. These include health care providers, community service providers, nonprofit organizations, policy makers, the public, academia, and pregnant and postpartum women. We will prepare, print, and share information notes about the project and its findings and share these among key stakeholders. We will also produce a 5-minute video documentary of the project that demonstrates what was done at each stage of the project and the ultimate outcomes. This project’s video documentary would be widely disseminated through various web-based and offline platforms. Our dissemination plan includes (1) peer-reviewed manuscripts, (2) peer-reviewed presentations at prestigious conferences, (3) presentations to stakeholders, (4) media releases, blog or website posts, (5) technical and research reports, and (6) presentations to postpartum women at the study facilities during postnatal care gatherings as well as community dissemination.

### Conclusions

This project will be the first of its kind to implement an integrated and comprehensive group postpartum care delivery model in Ghana, focused on the care of the mother in addition to the baby. Focused-PPC has the potential to change the postpartum care delivery model in Ghana and other countries in sub-Saharan Africa and beyond. Results from this implementation will be used to further refine and scale up the Focused-PPC model of postpartum care.

## Abbreviations

EPDS: Edinburgh Postnatal Depression Scale
Focused-PPC: Focused Postpartum Care
GHS: Ghana Health Service
MANOVA: multivariate ANOVA
PSS: Perceived Stress Scale
RHDS-NM: Readiness for Hospital Discharge Scale – New Mother Form
WHO: World Health Organization

## Data Availability

The data sets generated during or analyzed during this study are available from the corresponding author on reasonable request.
